# What are the applications of ChatGPT in healthcare: Gain or loss?

**DOI:** 10.1002/hsr2.1878

**Published:** 2024-02-14

**Authors:** Mahdieh Montazeri, Zahra Galavi, Leila Ahmadian

**Affiliations:** ^1^ Department of Health Information Sciences, Faculty of Management and Medical Information Sciences Kerman University of Medical Sciences Kerman Iran

**Keywords:** artificial intelligence, ChatGPT, language model, recommendations

## 1 CHATGPT IN HEALTHCARE

ChatGPT is a large language model developed by Open AI, based on the GPT (Generative Pre‐trained Transformer) architecture. It is an AI system that can understand natural language and generate coherent and contextually appropriate responses to a wide range of prompts and questions.^1^, ^2^ Today, this language model is in the center of attention in healthcare and other domains. Although this language model has several advantages in healthcare and medical education, it also has disadvantages. In this article, we discuss on these issues and provide some recommendations regarding this topic.

## 2 ADVANTAGES OF USING CHATGPT IN HEALTHCARE

ChatGPT can have several advantages in healthcare. First, personalized care. If the required information is available, ChatGPT can provide personalized care to patients by understanding their medical history, symptoms, and preferences. This can help healthcare providers to offer tailored treatment plans and improve patient outcomes.^3^ Second, availability. ChatGPT can be available 24/7, allowing patients who have access to this technology, to access healthcare information at any time. This is especially beneficial for patients who have urgent medical needs or live in remote areas.^4^ Third, improved efficiency. ChatGPT can help healthcare providers to improve their efficiency by automating routine tasks and repetitive actions, such as appointment scheduling and prescription refills. This can free up healthcare providers to focus on more complex tasks and provide high‐quality care.^5^ Forth, reduced costs. ChatGPT can help to reduce healthcare costs by minimizing the need for in‐person visits and hospitalizations. This can be especially beneficial for patients with chronic conditions who require ongoing care.^6^, ^7^ Fifth, patient education. If this technology is available, ChatGPT can be used to educate patients about their health conditions, medications, and treatment options. This can help patients to make informed decisions about their healthcare and improve their overall health outcomes. Moreover, this can be used to provide information to patients to help them comply with medical instructions.^4^ Sixth, improved communication. With its natural language processing capabilities, ChatGPT can facilitate better communication between patients and healthcare providers. It can help patients understand medical information and terminology, to find answers to their questions, and provide guidance on treatment options. Seventh, improved diagnosis. With its ability to understand complex medical data and patterns, ChatGPT can assist healthcare providers in making more accurate diagnoses. It can also help identify rare or difficult‐to‐diagnose conditions by analyzing patient data and symptoms. By providing differential diagnosis for asked signs and symptoms, helps providers to determine what action or physical examination should be done.^4^ Seventh, medical education. ChatGPT can serve as a valuable tool for medical education and training. It can provide healthcare students with access to a vast database of medical information and assist them in learning medical terminology and best practices.^8^ Eighth, mental health support. ChatGPT can assist patients with mental health conditions by providing them with support and resources. It can also help identify patients who may be at risk of developing mental health issues by analyzing their language and behavior.^9^ Ninth, health monitoring. ChatGPT can assist in monitoring and tracking patients’ health by analyzing data from wearable devices and patient reports. It can notify healthcare providers of any concerning changes in a patient's health, allowing for early intervention and treatment.^10^ Tenth, language assistance: ChatGPT can assist patients who speak different languages by providing translation services. This can help improve communication and understanding between patients and healthcare providers, which is essential for providing high‐quality care.^11^ Eleventh, health screening: By providing information, Chat GPT can be used for screening and early detection of conditions. It can provide information regarding the importance of regular screening tests and physical examinations.^12^, ^13^

## 3 DISADVANTAGES OF USING CHATGPT IN HEALTHCARE

Along with many advantages and improvements, ChatGPT also has limitations. These limitations include: First, limited knowledge. ChatGPT answers only as much as the data it is trained on. Therefore, it is not correct to claim that everything can be learned from it. Second, the inability to understand context. Sometimes ChatGPT is unable to understand the context beyond the information given. Its answers are limited to the information and context provided in the question or statement. Third, the possibility of bias. There is a risk of bias in ChatGPT answers based on the data used for training. Forth, privacy concerns. Users should be aware that their conversations with ChatGPT may be recorded and stored for research purposes. Fifth, lack of emotional intelligence. ChatGPT is unable to understand users’ emotions and cannot empathize with them. It is not possible to understand the awkward glance by a patient who seems to be avoiding to provide relevant information about the details of her's symptoms or the subjective sense of disease severity. Sixth, limited creativity, unable to produce creative content such as displaying understandable information for the patient. Seventh, inability to learn from experience. ChatGPT cannot learn from experience or adapt its behavior based on user feedback. Eighth, lack of awareness. Often users are unwilling to accept AI tools due to concerns about a lack of awareness and engagement with new of them.^14^ Ninth, dependence on Internet connection. ChatGPT functionality depends on an Internet connection, which can limit access in areas with poor connectivity. Tenth, inability to perform physical tasks. ChatGPT is unable to perform physical tasks or interact with the physical world. Eleventh, ChatGPT is also not suitable for patients who are not educated for this tool, especially for elders or people who lack of education.^15^ Twelfth, delay in updating knowledge. Given that ChatGPT's knowledge is limited to the period before 2021, it cannot currently be used as a reliable updated source.^4^ Thirteenth, inability to provide real‐time access to the latest data. Since ChatGPT does not have real‐time access to the latest data, information currency is a concern.^16^ Fourteenth, complexity of answers. ChatGPT answers vary according to the complexity of the questions. When a complex question is asked, the answer will be complex.^17^

Some of these restrictions in the field of health can have irreparable damages. For example, with limited knowledge of ChatGPT, if the patient performs treatment based on ChatGPT responses and that treatment is not suitable for the person, it can worsen the condition for him. It is better for people to be aware of the limitations of this robot and know that they should not perform any action without checking by experts. Regarding privacy, given the importance of preserving health data, users should know where their data is stored and for what purposes it will be used.

## 4 RECOMMENDATIONS FOR USING CHATGPT IN HEALTHCARE

To have a safer use of ChatGPT, one can consider the following recommendations: First, use ChatGPT for informational purposes only. ChatGPT can provide helpful information about medical conditions, treatments, and medications, but it's important to remember that this information should not be used to diagnose or treat a medical condition. Always consult with a qualified healthcare professional for medical advice. Second, be specific in your questions. When asking ChatGPT a question, try to be as specific as possible. This will help ChatGPT provide more accurate and relevant information. Third, use ChatGPT to augment, not replace, human healthcare providers. While ChatGPT can provide valuable support and guidance to patients, it is important to remember that it should not replace human healthcare providers. Instead, it should be used to augment their work and provide patients with additional support and information. Fourth, verify information. ChatGPT is a language model that generates responses based on patterns in the data it was trained on. While ChatGPT strives to provide accurate information, it's always a good idea to verify any information you receive with a qualified healthcare professional or reputable source. Fifth, protect your privacy. When using ChatGPT, be mindful of the information you share. Avoid sharing personal health information that could compromise your privacy or security.

By following these recommendations, healthcare providers can ensure that ChatGPT is used effectively and responsibly in the healthcare industry.

## 5 CONCLUSIONS

The use of artificial intelligence systems technology, including the widespread use of ChatGPT in healthcare, is inevitable. Therefore, to use this technology ethically, safely and responsibly, it is necessary to establish appropriate guidelines and regulations with the participation of all stakeholders. If implemented correctly and appropriately, CHATGPT has the potential to accelerate innovation in healthcare.

## AUTHOR CONTRIBUTIONS

**Mahdieh Montazeri**: conceptualization; writing—original draft; writing—review & editing. **Zahra Galavi**: conceptualization; writing—original draft. **Leila Ahmadian**: conceptualization; writing—review & editing.

## CONFLICT OF INTEREST STATEMENT

The authors declare no conflicts of interest.

## ETHIC STATEMENT

This research was approved by the ethics committee of Kerman University of Medical Sciences with the ethics code IR.KMU.REC.1402.233.

## TRANSPARENCY STATEMENT

The lead author Leila Ahmadian affirms that this manuscript is an honest, accurate, and transparent account of the study being reported; that no important aspects of the study have been omitted; and that any discrepancies from the study as planned (and, if relevant, registered) have been explained.

## Data Availability

The authors confirm that the data supporting the findings of this study are available within the article or its supplementary materials.
